# K15 promoter-driven enforced expression of NKIRAS exhibits tumor suppressive activity against the development of DMBA/TPA-induced skin tumors

**DOI:** 10.1038/s41598-021-00200-1

**Published:** 2021-10-19

**Authors:** Kenji Tago, Satoshi Ohta, Chihiro Aoki-Ohmura, Megumi Funakoshi-Tago, Miho Sashikawa, Takeshi Matsui, Yuki Miyamoto, Taeko Wada, Tomoyuki Oshio, Mayumi Komine, Jitsuhiro Matsugi, Yusuke Furukawa, Mamitaro Ohtsuki, Junji Yamauchi, Ken Yanagisawa

**Affiliations:** 1 Division of Structural Biochemistry, Department of Biochemistry, School of Medicine, Jichi Medical University, 3311-1 Yakushiji, Shimotsuke-shi, Tochigi 329-0498 Japan; 2 Division of Hygienic Chemistry, Faculty of Pharmacy, Keio University, 1-5-30 Shibakoen, Minato-ku, Tokyo 105-8512 Japan; 3 Department of Dermatology, School of Medicine, Jichi Medical University, 3311-1 Yakushiji, Shimotsuke-shi, Tochigi 329-0498 Japan; 4 Laboratory for Evolutionary Cell Biology of the Skin, School of Bioscience and Biotechnology, Tokyo University of Technology, 1404-1 Katakura, Hachioji, Tokyo 192-0982 Japan; 5 Department of Pharmacology, National Research Institute for Child Health and Development, Setagaya, Tokyo 157-8535 Japan; 6 Division of Stem Cell Regulation, Center for Molecular Medicine, Jichi Medical University, 3311-1 Yakushiji, Shimotsuke-shi, Tochigi 329-0498 Japan; 7 Laboratory of Molecular Neuroscience and Neurology, Tokyo University of Pharmacy and Life Sciences, Hachioji, Tokyo 192-0392 Japan

**Keywords:** Oncogenes, Cancer, Tumour-suppressor proteins

## Abstract

NKIRAS1 and NKIRAS2 (also called as κB-Ras) were identified as members of the atypical RAS family that suppress the transcription factor NF-κB. However, their function in carcinogenesis is still controversial. To clarify how NKIRAS acts on cellular transformation, we generated transgenic mice in which NKIRAS2 was forcibly expressed using a cytokeratin 15 (K15) promoter, which is mainly activated in follicle bulge cells. The ectopic expression of NKIRAS2 was mainly detected in follicle bulges of transgenic mice with NKIRAS2 but not in wild type mice. K15 promoter-driven expression of NKIRAS2 failed to affect the development of epidermis, which was evaluated using the expression of K10, K14, K15 and filaggrin. However, K15 promoter-driven expression of NKIRAS2 effectively suppressed the development of skin tumors induced by treatment with 7,12-dimethylbenz(a)anthracene (DMBA)/12-*O*-tetradecanoylphorbol 13-acetate (TPA). This observation suggested that NKIRAS seemed to function as a tumor suppressor in follicle bulges. However, in the case of oncogenic HRAS-driven cellular transformation of murine fibroblasts, knockdown of NKIRAS2 expression drastically suppressed HRAS-mutant-provoked cellular transformation, suggesting that NKIRAS2 was required for the cellular transformation of murine fibroblasts. Furthermore, moderate enforced expression of NKIRAS2 augmented oncogenic HRAS-provoked cellular transformation, whereas an excess NKIRAS2 expression converted its functional role into a tumor suppressive phenotype, suggesting that NKIRAS seemed to exhibit a biphasic bell-shaped enhancing effect on HRAS-mutant-provoked oncogenic activity. Taken together, the functional role of NKIRAS in carcinogenesis is most likely determined by not only cellular context but also its expression level.

## Introduction

It is well understood that the RAS family includes critical GTPases in the regulation of cell proliferation, survival, and cell differentiation in a wide variety of mammalian cells^1,2^. Among the classical RAS family, three members HRAS, KRAS and NRAS exhibit high structural similarity and regulate similar effectors such as RAF family protein kinase and phosphatidyl inositol-3 kinase (PI3K), upstream of the extracellular signal-regulated kinase (ERK) and Akt pathways, respectively^3–5^. Numerous studies have reported the presence of atypical Ras-like GTPases and clarified their biological functions. RRAS shows 55% amino acid identity with the classical Ras family and interacts and regulates the activity of RAF kinase, Bcl-2, RAL-GEF, and PI3K^6–10^. RAP and RHEB are other members of the Ras family. RAP regulates cell adhesion mediated by cadherin and integrin^11–13^, and RHEB is involved in the activation of mammalian target of rapamycin complex 1 (mTORC1)^14^. Two structurally similar proteins, DIRAS1 and DIRAS2 possess low GTP-hydrolysis activity and are mainly present as a GTP-bound form without any mitogenic stimulation^15^. A DIRAS homologue is also expressed in nematodes, and it was reported that enforced expression of DIRAS homologue in neuronal cells modulated synaptic activity^16^.

NKIRAS1 and NKIRAS2 (NKIRAS1/2) were originally identified as members of the nuclear factor of kappa light polypeptide gene enhancer in B-cells inhibitor (IκB)-interacting RAS superfamily. NKIRAS1/2 were reported to negatively regulate tumor necrosis factor alpha (TNFα)-induced nuclear factor kappa-light-chain-enhancer of activated B cells (NF-κB) activation^17^. Unlike the classical Ras family, NKIRAS lacks a carboxy-terminal CAAX motif, which harbors the amino acid sequence for lipid modification. In addition, the primary structure in the switch 1 and switch 2 domains of NKIRAS decreases the GTP-hydrolysis activity of NKIRAS, suggesting that NKIRAS probably exists as a constitutive GTP-bound form in cells^17^. Indeed, we previously demonstrated that NKIRAS predominantly bound to GTP without any agonistic stimulation such as epidermal growth factor (EGF) or platelet-derived growth factor (PDGF)^18^. We also showed that the cellular localization of NKIRAS is regulated by its GTP/GDP-binding state. NKIRAS interacted with a complex including IκB alpha (IκBα) and NF-κB or IκB beta (IκBβ) alone, resulting in the suppression of their proteasomal degradation^17^. Furthermore, another report showed that NKIRAS also inhibited the phosphorylation of IκBβ by the IκB kinase (IKK) complex with its both GTP- and GDP-bound forms^19^. We also reported that NKIRAS exhibits higher binding affinity with the RelA subunit of the NF-κB complex rather than IκB proteins. This interaction is critical for the inhibition of NF-κB, because NKIRAS binds to the RelA subunit and inhibits phosphorylation of this subunit at Ser-276^18^. Phosphorylation of the RelA subunit at Ser-276 is essential for the transcriptional activation of NF-κB and accumulated in Ras mutant-driven colorectal cancer tissues^20,21^.

Oeckinghaus and colleagues generated Nkiras1- and Nkiras2-deficient mice^22^. Although both Nkiras1^−/−^ mice and Nkiras2^−/−^ mice developed normally, double knockout mice (Nkiras1^−/−^/Nkiras2^−/−^) exhibited perinatal lethality. This perinatal lethality was rescued using a TNFα^−/−^ background. Furthermore, they reported that NKIRAS functioned as a tumor suppressor mediated by inhibition of RALA small GTPase. It was reported that RALA may be involved in the activation of phospholipase D and mTORC1^23,24^, and these two signaling cascades were reported to be involved in carcinogenesis^25–27^.

Keratins (also called cytokeratins) are intermediate filament proteins commonly expressed in epithelial cells^28^. Human keratins include 54 members. Among them, K10 is expressed in stratum spinosum and granular layer, and K14 is expressed in basal layer^29^. It is well understood that Keratin 15 (K15) is mainly expressed in skin follicle cells, and also detectable in interfollicular epidermis^29^.

To obtain insights about the functional role of NKIRAS for carcinogenesis in vivo, we generated transgenic mice in which NKIRAS2 was forcibly expressed using the K15 promoter. Using these transgenic mice, we attempted to clarify how NKIRAS affects oncogenic signals in vivo. In addition, we also investigated the function of NKIRAS in the cellular transformation of murine fibroblasts and compared the functional role of NKIRAS in carcinogenesis in two experimental systems including transgenic mice and cultured cells.

## Materials and methods

### Reagents

Anti-FLAG antibody (M2) was purchased from Sigma-Aldrich (St. Louis, MO). Anti-HRAS and anti-β-actin antibodies were purchased from Santa Cruz Biotech (Dallas, TX). Anti-NKIRAS2 antibody was obtained as described previously^18^. Anti-cytokeratin 10, 14, 15 (K10, K14 and K15) and anti-filaggrin antibodies were obtained from Cell Signaling Technology (Danvers, MA). Anti-phospho- and total-ERK, JNK, and Akt antibodies were also from Cell Signaling Technology. Other reagents were purchased from Sigma-Aldrich and Nacalai Tesque (Kyoto, Japan).

### Generation of transgenic mice

**Figure 1:**
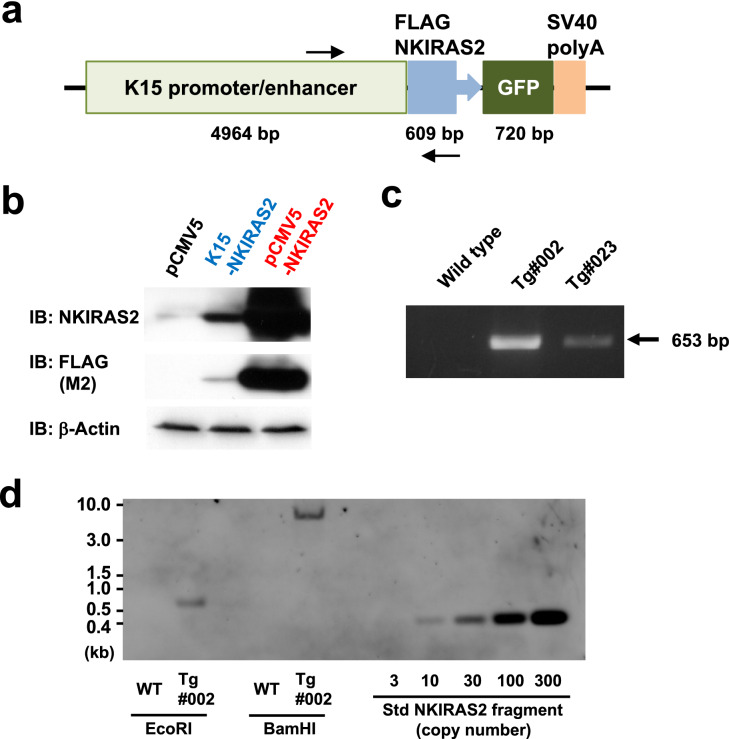
Development of NKIRAS2 transgenic mice. (**a**) The structure of the transgene for expressing NKIRAS2 using the K15 promoter is shown. GFP is the landmark for inserted transgene; however, the protein is not expressed. The arrows indicate the position of designed primers to detect the NKIRAS2 transgene. (**b**) Using HEK293T cells, the expression of NKIRAS2 from the constructed NKIRAS2 transgene was confirmed by immunoblotting. As negative and positive control, pCMV5 and pCMV5-NKIRAS2 was utilized, respectively. (**c**) Using two lines of developed transgenic mice, #002 and #023, the presence of the transgene for NKIRAS2 was confirmed by PCR using genomic DNA samples from wild type and transgenic mice as templates. (**d**) To determine the copy number of NKIRAS2 transgene, southern blot analysis was performed. Blot for transgenic mice #023 was shown in Supplementary Fig. 1.

Genetically modified/unmodified mice were cared for in accordance with a protocol approved by the Japanese National Research Institute for Child Health and Development Animal Care Committee and were monitored by the Laboratory Animal Facility of the Japanese National Research Institute for Child Health and Development. To construct the transgenic vector for NKIRAS2, a cDNA of amino-terminal FLAG-tagged NKIRAS2 was inserted into the *Eco*RI sites of a K15-EGFP vector, which was deposited in addgene (Watertown, MA) by Dr. George Cotsarelis^30^. In the constructed vector, the expression activity of EGFP is inactivated by insertion of NKIRAS2 cDNA (Fig. 1a). The vector was digested with *Afl*II and *Bgl*II, and then the transgene including NKIRAS2 cDNA was isolated, and purified. The transgene was injected into fertilized C57BL/6JJms oocytes. Transgenic founder mice and established transgenic mice were identified using tail genomic PCR with specific primers for transgenes as described below.

- Forward: 5′-AGTCTTTTCAGCGTGTG-3′
- Reverse: 5′-TGCTGGCCAAGTAGACA-3′

Transgenic founders were mated with wild type C57BL/6JJms mice and the littermates were used for experiments. Transgenic mice as well as their non-transgenic littermates were fertile in standard breeding conditions. Male mice were used for experiments with 7,12-dimethylbenz(a)anthracene (DMBA)/12-*O*-tetradecanoylphorbol 13-acetate (TPA)-induced papilloma formation.

### Southern blotting analysis

Thirty µg of genomic DNAs were digested with EcoRI or BamHI, and separated by 1% agarose-TAE electrophoresis. The separated genomic DNAs were transferred onto Hybond-N^+^ nitrocellulose membrane (Cytiva, Marlborough, MA) with SSC solution. PCR product of human NKIRAS2 was labeled with digoxigenin by using Kit (Roche diagnostics, Indianapolis, IN), and utilized as probe. The hybridized DNA probe was detected by the incubation with HRP-conjugated anti-digoxigenin antibody, and visualized by ECL (Cytiva).

### Preparation of a keratinocyte-enriched cellular fraction

From newborn mice, skin was isolated surgically and treated with 1,500 PU/mL Dispase (Fujifilm, Tokyo, Japan) at 37 °C for 20 min. Then, the epidermis was physically peeled from the treated skin. The isolated epidermis was washed with phosphate buffered saline (PBS) containing 1 mM CaCl_2_ and treated with triple protease (Invitrogen, Carlsbad, CA) at 37 °C for 20 min. The treated epidermis was transferred to a fresh 1 mL aliquot of triple protease, and the keratinocyte-included cellular fraction was harvested. The prepared cells were washed with PBS and utilized for analysis including reverse transcription-polymerase chain reaction (RT-PCR).

### RT-PCR

To prepare total RNA from the prepared keratinocyte-containing cellular fraction, cells were suspended with 1 mL of TRI-reagent (Sigma-Aldrich). Then, 0.2 mL of chloroform was added and mixed by vortexing for 15 s. After centrifugation, the obtained upper layer was transferred to a fresh tube, and the RNA fraction was precipitated by the addition of 3 volumes of ethanol and recovered by centrifugation. Then, contaminating genomic DNA was removed by treatment with DNase I (Qiagen, Germantown, MD) and phenol/chloroform extraction. After ethanol precipitation, the prepared RNA fraction was utilized for cDNA synthesis using reverse-transcription with ReverTra Ace (TOYOBO, Osaka, Japan). To detect the expression of the transgene-derived NKIRAS2 expression, PCR using the synthesized cDNA was performed, and amplified PCR fragments were detected by agarose electrophoresis and ethidium bromide staining. Sequences of the utilized PCR primers were: 5′-TGGGGCCGAACTGCCCCGA-3′ and 5′-CGTCATCTTGCTGGCCAAGTAGACA-3′ for human NKIRAS2, 5′-GGAAGAGATCCGGGACAAA-3′ and 5′-TGTCAATCTCCAGGACAACG-3′ for murine K15, 5′-ACCACAGTCCATGCCATCAC-3′ and 5′-TCCACCACCCTGTTGCTGTA-3′ for murine GAPDH.

### DMBA/TPA-induced papilloma formation

Prior to chemical treatment, 6-week-old mice had the hair on their back shaved. Next day, 100 µL of 0.1% (w/v) DMBA (Fujifilm) in acetone solution was applied onto the shaved area. After 1 week, the mice were treated with 100 µL of 0.1% (w/v) TPA (Fujifilm) in acetone solution onto the DMBA-treated area. Treatment with TPA was performed twice weekly, 3–4 days apart. The formation of tumors was visible after 6 months of treatment with DMBA/TPA.

### Immunofluorescence analysis

Isolated skin was fixed with 2% formaldehyde in PBS for 1 h. After fixation, skin was soaked in 20% sucrose in PBS for 2–3 days. The skin pieces were embedded in Tissue-Tek reagent (Sakura Finetechnical, Tokyo, Japan) by freezing at − 80 °C until use. The samples were used for the preparation of frozen skin tissue Sect. (0.45 mm) using a Cryostat (Thermofisher). The prepared sections were sequentially stained with the indicated primary antibodies and Alexa Fluor 488-conjugated goat anti-rabbit antibody (Invitrogen). In the case of staining for the FLAG tag, an M2 antibody directly fused with FITC (Sigma-Aldrich) was used. The nucleus was visualized by the addition of DAPI (Vector Laboratories, Burlingame, CA). Samples were analyzed using an FSX100 fluorescence microscope (Olympus, Tokyo, Japan).

### Immunohistochemistry

Isolated skins were fixed with PBS containing 4% formaldehyde. They were postfixed with 4% paraformaldehyde, replaced with 20% sucrose, and embedded in paraffin. The embedded skin samples were utilized for the preparation of paraffin sections. The prepared paraffin sections were activated in an autoclave. Microtome sections were stained with hematoxylin–eosin (H&E) or incubation with indicated antibodies. H&E staining was outsourced to Hirota surgical pathology institute Inc. and Kyoto byori Inc.

### Immunoblotting

Cells were lysed with RIPA buffer (10 mM sodium phosphate (pH 7.2), 150 mM NaCl, 3 mM MgCl_2_, 2 mM EDTA, 1% (v/v) NP-40, 1% (w/v) sodium deoxycholate, 20 mM β-glycerophosphate, 100 µM sodium orthovanadate, and protease inhibitors cocktail (Sigma-Aldrich)), and briefly sonicated on ice. Then, debris was removed by centrifugation at 13,000 rpm for 10 min at 4 °C. After mixing with Laemmli’s buffer, cell lysates were separated by sodium dodecyl sulfate–polyacrylamide gel electrophoresis, and transferred to polyvinylidene difluoride (PVDF) membrane (Merck Millipore, Burlington, MA). PVDF membranes with protein lysates were sequentially incubated with PBS including 0.05% (v/v) Tween-20 including indicated antibodies and secondary antibodies conjugated with horseradish peroxidase. The analyzed proteins were visualized by reaction of the peroxidase with ECL (GE Healthcare, IL, Chicago).

### Cell culture and retroviral infection

NIH-3T3 fibroblasts and HEK293T cells were maintained in Dulbecco's modified Eagle's medium supplemented with 10% fetal calf serum, 2 mM glutamine, and 100 units each of penicillin and streptomycin (Sigma-Aldrich). To prepare conditioned medium containing retroviruses, HEK293T cells were transfected with retroviral backbone plasmids and helper plasmids such as pE-Eco and pGP (TAKARA BIO, Shiga, Japan). To prepare the retroviruses for enforced expression of HRAS (G12V) or NKIRAS2, pBabePuro or pMSCV-ires-Puro was utilized as a retroviral back-born plasmid. To silence the expression of endogenous NKIRAS2, pSUPER-retro-Puro (Oligoengine, Seattle, WA) harboring the sequences for sh-RNAs was utilized. Retroviruses were produced into the conditioned medium, and then the conditioned medium was collected several times, and stood in ice until infection. NIH-3T3 cells (cell number per 60 mm-diameter dish) were infected with retroviruses mixed with 1 µg/mL polybrene (Sigma-Aldrich). For the infection with retrovirus harboring HRAS (G12V), the retrovirus was diluted tenfold, according to previous optimization^31^. Next day, the infected cells were selected using 7.5 µg/mL puromycin (Invivogen, San Diego, CA) for 3 days. After puromycin selection, the infected cells were utilized for immunoblotting analysis and colony formation assays in soft agar medium.

### Colony formation assay in soft agar media

The infected NIH-3T3 cells were collected by trypsinization. Then, 1 × 10^4^ cells from each sample were mixed with complete DMEM containing 0.3% noble agar (Invitrogen), and seeded onto hardened complete DMEM containing 0.5% noble agar. After 2–3 weeks, colony formation was observed and their number was counted.

### Analysis of data

All experiments were performed individually three times, and representative data shown. In graphs, error bars indicate standard deviation (S.D., n = 3), and the results of calculations of independent t-tests are shown. Differences were considered to be significant for values of *p* < 0.01. For immunoblot analysis in this manuscript, uncropped data are shown in Supplementary figures (2).

### ARRIVE guidelines

All experiments were performed according to the ARRIVE guideline. All the institutional guidelines and regulations for animal experiments were followed in the Methods section in addition to the approval statement that is provided.

## Results

### Generation of NKIRAS2 transgenic mice

To investigate whether NKIRAS affects skin development and tumor formation, we generated transgenic mice forcibly expressing NKIRAS2 driven by the K15 gene promoter (Fig. 1a). We inserted the cDNA of FLAG-tagged human NKIRAS2 (FLAG- NKIRAS2) into the K15-EGFP vector^30^. To confirm that the K15 promoter can induce the expression of NKIRAS2, immunoblotting analysis was performed with lysates of HEK293T cells transfected with the constructed vector (Fig. 1b). The K15 promoter successfully induced the expression of FLAG-tagged human NKIRAS2, which was detected using both the anti-NKIRAS2 antibody and anti-FLAG M2 antibody. However, its expression level was lower than the expression of NKIRAS2 driven by the CMV promoter (Fig. 1b). Then, the DNA fragment including the transgene for NKIRAS2 was injected into fertilized mouse oocytes. We successfully obtained two individual lines of transgenic mice named NKIRAS2 transgenic mice #002 and #023, which were confirmed to include the transgene by genomic PCR (Fig. 1c). To determine the copy number of NKIRAS2 transgene, southern blot analysis was performed. The transgenes of FLAG-NKIRAS2 inserted into genomic DNA were quantified as about 25 and 5 copies in transgenic mice #002 and #023, respectively (Fig. 1d and Supplementary Fig. 1). These transgenic mice appeared healthy and normally bred.

### K15 promoter-driven enforced expression of NKIRAS2 in follicle cells

**Figure 2:**
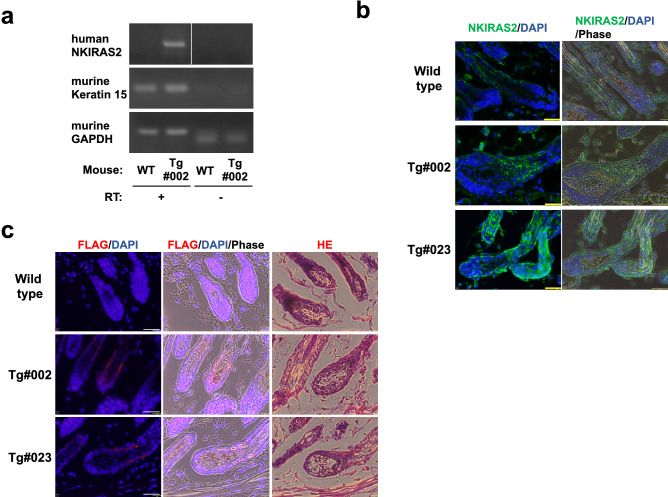
Expression of ectopic NKIRAS2 driven by the K15 promoter in follicle bulges. (**a**) To detect the expression of transgenic NKIRAS2 mRNA, total RNA was prepared from a keratinocyte-enriched cellular fraction of wild type and NKIRAS2 #002 transgenic mice, and then utilized for RT-PCR with/without reverse transcription (RT). To confirm the presence of the cellular population from follicle bulges, the expression of K15 mRNA was examined. The expression of transgenic NKIRAS2 is shown by RT-PCR using specific primers for human NKIRAS2. GAPDH is shown as an internal control. (**b**) Ectopic expression of NKIRAS2 driven by the K15 promoter in follicle bulges of wild type and two lines of transgenic mice is shown using immunofluorescence analysis with an anti-NKIRAS2 antibody (green). Nuclei of all cells in the epidermal section were stained with DAPI (blue). Scale bar = 153 µm (**c**). To confirm that exogenous NKIRAS2 was expressed, NKIRAS2 was stained with an anti-FLAG antibody (red). To show follicular bulge, H&E staining was performed. Scale bar = 32 µm.

To investigate mRNA expression of NKIRAS2 from the transgene, we prepared total RNA from epidermis of newborn transgenic mice and wild type mice. We prepared a keratinocyte-enriched cellular fraction from epidermis, because follicle bulges may be included in the epidermis. To remove contamination with transgene for NKIRAS2 in genomic DNA, total RNA was treated with DNase I. Then, total RNA was utilized for RT-PCR analysis. We first analyzed the expression of K15 to show that the prepared keratinocyte-enriched cellular fraction contained follicle bulges prepared from transgenic mice. As shown in Fig. 2a, the expression of K15 was detected in both transgenic mice #002 and wild type mice. Next, we designed PCR primers to amplify the cDNA of only human NKIRAS2 but not endogenous murine Nkiras2, and then performed RT-PCR. The expression of human NKIRAS2 was detected in only total RNA extracted from transgenic mice #002 but not from wild type mice (Fig. 2a). We also attempted to detect the expression of NKIRAS2 in the transgenic mice #023, however its mRNA was not detected by RT-PCR. By performing immunofluorescence analysis for skin sections of newborn mice, we next analyzed the expression of NKIRAS2 in the skin of transgenic mice and wild type mice. Since endogenous Nkiras2 is ubiquitously expressed^18^, it was hard to distinguish ectopic NKIRAS2 and endogenous Nkiras2 (Supplementary Fig. 2). However, when using higher magnification (40-fold), both transgenic mice #002 and #023 moderately showed higher intensities of NKIRAS2 expression in their hair follicle (Fig. 2b). Because the NKIRAS2 transgene was tagged with FLAG at its amino terminus, we next utilized an anti-FLAG antibody (M2) for immunostaining analysis to detect the exogenous expression of NKIRAS2. As shown in Fig. 2c and Supplementary Fig. 3, the anti-FLAG antibody (M2) specifically detected the expression of exogenous FLAG-tagged NKIRAS2 in the follicle bulges of both NKIRAS2 transgenic mice #002 and #023 but not wild type mice. Anti-FLAG antibody also stained epidermis of transgenic mice, however the epidermis of wild type mice was comparably stained, suggesting that the epidermis seems to be non-specifically stained with anti-FLAG antibody. To confirm the structure of follicle bulge, H&E staining was also performed (Fig. 2c). Taken together, in the generated transgenic mice #002 and #023, FLAG-tagged NKIRAS2 was specifically expressed in follicle bulge.

### Effect of K15 promoter-driven NKIRAS2 on the development of skin

**Figure 3:**
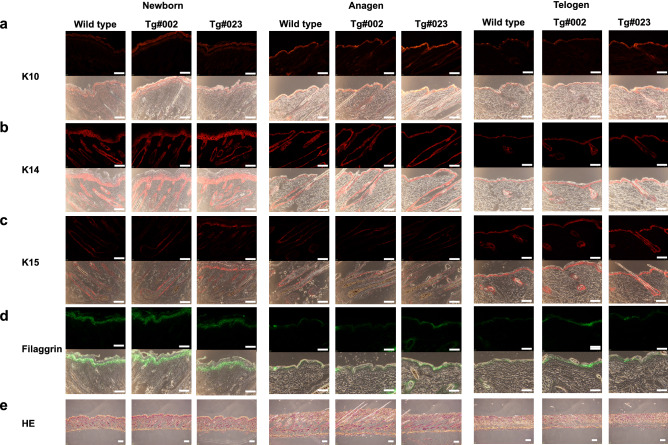
Effect of ectopic expression of NKIRAS2 on the development of epidermal tissue. (**a**–**d**) To test the effect of ectopic expression of NKIRAS2 on the development of epidermal tissue, epidermal sections from wild type, NKIRAS2 #002 and #023 transgenic mice were stained with an anti-K10, K14, K15 and filaggrin antibody (K10, K14 and K15 were stained with red, and filaggrin was stained with green). Photographs of phase contrast were also taken, and were merged with the photograph of immunofluorescence analysis. Scale bar = 32 µm. (**e**) To show the development of skin, frozen sections of skin prepared from wild type and transgenic mice #002 and #023 were stained with H&E. Scale bar = 311 µm.

Next, we investigated the effect of the enforced expression of NKIRAS in follicle bulge on the development of skin by performing the immunofluorescence analysis for several skin markers such as K10, K14, K15 and filaggrin. Furthermore, we analyzed them at three stages, newborn mice (1 or 2 days after birth), anagen (5-weeks after birth) and telogen (7-weeks after birth), respectively. It is well understood that K10 is expressed in stratum spinosum and granular layer^32^. K14 is also well known as marker protein for basal layer^32^. The enforced expression of NKIRAS2 did not affect the expression pattern of K10 and K14 in newborn mice skin, anagen and telogen skin (Fig. 3a, b, supplementary Figs. 4 and 5). Furthermore, we analyzed the expression of K15. K15 was known as marker protein of follicle bulge stem cells^29^, however also detected in interfollicular epidermis as reported by several investigations^33,34^. As shown in Fig. 3c and Supplementary Fig. 6, the expression of K15 was detected in follicle bulges and epidermis of wild type mice in newborn mice, anagen and telogen skin. Then, we analyzed the expression of K15 in transgenic mice, and the intensity and pattern of K15 expression in transgenic mice were comparable with its expression in wild type. We also analyzed the expression of filaggrin. The expression of filaggrin in the epidermis was comparably detected in both of transgenic and wild type mice (Fig. 3d and Supplementary Fig. 7). Furthermore, we also performed H&E staining of each sample, and any alterations of structure of skin were not observed (Fig. 3e). These observations strongly suggested that the enforced expression of NKIRAS2 with the K15 promoter did not affect the development of follicle bulges and epidermis.

### Enforced expression of NKIRAS2 in follicle bulges suppresses DMBA/TPA-induced papilloma formation

**Figure 4:**
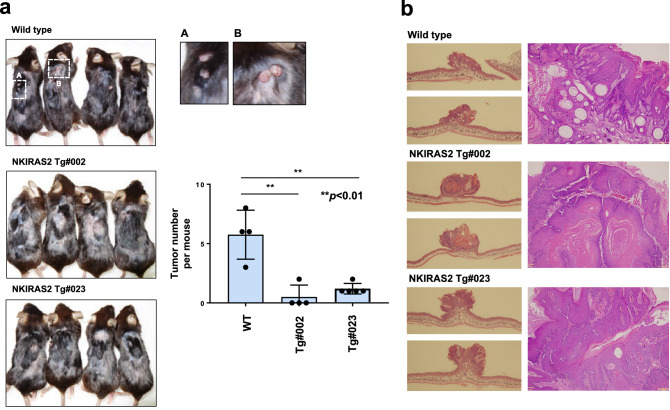
Effect of ectopic expression of NKIRAS2 on DMBA/TPA-induced papilloma development. (**a**) After sequential treatment with DMBA and TPA for 6 months, papilloma developed on skin of wild type and NKIRAS2 transgenic mice, #002 and #023 are shown in the photograph. The number of tumors is shown in the graph. The graph shows means with the error bars indicating S.D. (n = 3), and **indicates *p* < 0.01. (**b**) Paraffin-embedded sections of the epidermis with developed papilloma were prepared and stained with H&E.

It is well established that sequential treatment with DMBA and TPA on murine skin causes tumor formation^35^. Therefore, we investigated whether the enforced expression of NKIRAS2 driven by the K15 promoter affected tumor formation caused by treatment with DMBA/TPA. After 6 months of treatment with DMBA/TPA on murine skin, the formation of several tumors on the skin of wild type mice was observed (Fig. 4a). On the other hand, only low numbers of tumors formed on DMBA-TPA-treated skin in both lines of NKIRAS2 transgenic mice, suggesting that K15 promoter-driven ectopic expression of NKIRAS2 suppressed DMBA/TPA-induced tumor formation (Fig. 4a). Next, we prepared paraffin-embedded sections from isolated skin, on which tumors had formed on wild type mice and two lines of NKIRAS2 transgenic mice. Then, we stained these sections with H&E (Fig. 4b). These observations suggested that the enforced expression of NKIRAS2 driven by the K15 promoter suppressed the development of papilloma caused by treatment with DMBA/TPA. In conclusion, NKIRAS2 seems to function as a tumor suppressor in follicle bulges.

### Endogenous NKIRAS2 is required for oncogenic Ras-mutant-driven cellular transformation of murine fibroblasts

**Figure 5:**
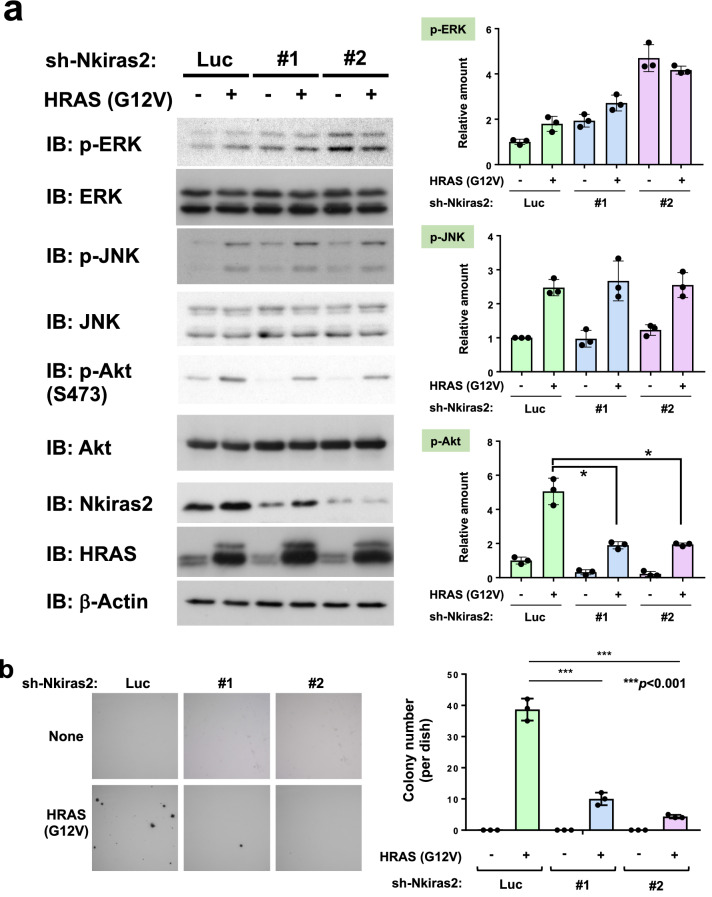
Effect of knockdown of Nkiras2 on HRAS (G12V)-provoked cellular transformation. (**a**) NIH-3T3 cells were infected with the indicated combination of retroviruses, and their lysates were utilized for immunoblotting analysis. To decrease Nkiras2 expression, two types of retroviruses harboring sh-RNA against murine Nkiras2 (called as sh-Nkiras2#1 and #2, also indicated as #1 and #2, respectively) were used. As control knockdown, retrovirus including sh-luciferase (sh-Luc; described as Luc) was utilized. The knockdown efficiency was examined using immunoblotting analysis. The effects of Nkiras2 knockdown on HRAS (G12V)-induced phosphorylation of ERK, JNK, and Akt are shown. As an internal control, the total amounts of ERK, JNK, and Akt are shown. The intensities of protein phosphorylation were shown in the graphs. (**b**) The infected NIH-3T3 cells as in (**a**) were utilized for a soft agar colony formation assay. The number of colonies is shown in the graph. All experiments were performed individually three times, and representative data shown in the graph. The graph shows means with the error bars indicating S.D. (n = 3), and * and ***indicates *p* < 0.01 and 0.001, respectively.

In a previous study, Oeckinghaus and colleagues reported that κB-Ras functions as a tumor suppressor, which diminishes RAL GTPase-mediated oncogenic signaling^22^. Furthermore, Beel et al. also showed that κB-Ras functions similarly as a tumor suppressor against the development of pancreatic adenocarcinoma in humans^36^. Our experimental results using NKIRAS2 transgenic mice were in agreement with their reports. However, when looking at Kaplan–Meier plots for several cases of human cancers, it has been suggested that the correlation between the tumor malignancy and the expression level of NKIRAS1/2 is still controversial (for NKIRAS1: https://www.proteinatlas.org/ENSG00000197885-NKIRAS1/pathology; for NKIRAS2: https://www.proteinatlas.org/ENSG00000168256-NKIRAS2/pathology). To gain further insights to clarify the relationship between tumor malignancy and NKIRAS expression, we analyzed the function of NKIRAS in the cellular transformation provoked by the oncogenic Ras mutant. As shown previously, Nkiras2 but not Nkiras1 is expressed in murine NIH-3T3 fibroblast cells^18^. Therefore, we designed retroviral vectors harboring sh-RNA against murine Nkiras2. As shown in Supplementary Fig. 8a, the expression of Nkiras2 was successfully silenced by sh-RNA #1 and #2 with more than 50% and 70% knockdown efficiencies, respectively. Using these sh-RNA retroviruses, we analyzed the effect of silencing of Nkiras2 expression on HRAS (G12V)-provoked cellular transformation by using NIH-3T3 cells. As shown in Fig. 5a and Supplementary Fig. 9, HRAS (G12V) was well expressed, and infection of NIH-3T3 cells with two kinds of retroviruses including sh-RNA against murine Nkiras2 caused effective reductions in Nkiras2 protein expression. However, comparing with RT-PCR analysis shown in Supplementary Fig. 8a, knockdown efficiency of Nkiras2 protein seemed to be lower. Although we do not have perfect explanation for this problem, we considered that NKIRAS2 is stable protein, and this character could cause the delay of degradation of NKIRAS protein. Then, using these infected cells, we performed soft agar colony formation assay. As shown in Fig. 5b, HRAS (G12V) induced the formation of colonies in soft agar media. Furthermore, HRAS (G12V) augmented the expression of Nkiras2 protein, however the expression of Nkiras2 mRNA was reduced by HRAS (G12V) (Supplementary Fig. 8b). Next, we investigated the effect of Nkiras2 knockdown on HRAS (G12V)-provoked cellular transformation. The knockdown of Nkiras2 expression drastically suppressed oncogenic HRAS mutant-provoked cellular transformation (Fig. 5b). We also analyzed the effects of Nkiras2 knockdown on the activation of ERK, JNK, and Akt, which were reported to corelate well with Ras-provoked carcinogenesis^37–40^. Strikingly, the knockdown of Nkiras2 effectively suppressed Ras-induced Akt phosphorylation at Ser473, which was reported to be induced by the activation of mTORC2^41,42^. In addition, the suppression of Akt phosphorylation by Nkiras2 knockdown was also observed in the condition without the enforced expression of oncogenic HRAS mutant. On the other hand, the activation of ERK and JNK was not suppressed by knockdown of Nkiras2 (Fig. 5a). Especially, the activation of ERK was effectively enhanced by Nkiras2 knockdown in the condition with/without the expression of HRAS (G12V). These observations suggested that Nkiras is required for oncogenic Ras-mutant-driven cellular transformation of murine fibroblasts.

### Functional direction of NKIRAS2 for tumorigenesis is altered by the cellular context and its expression level

**Figure 6:**
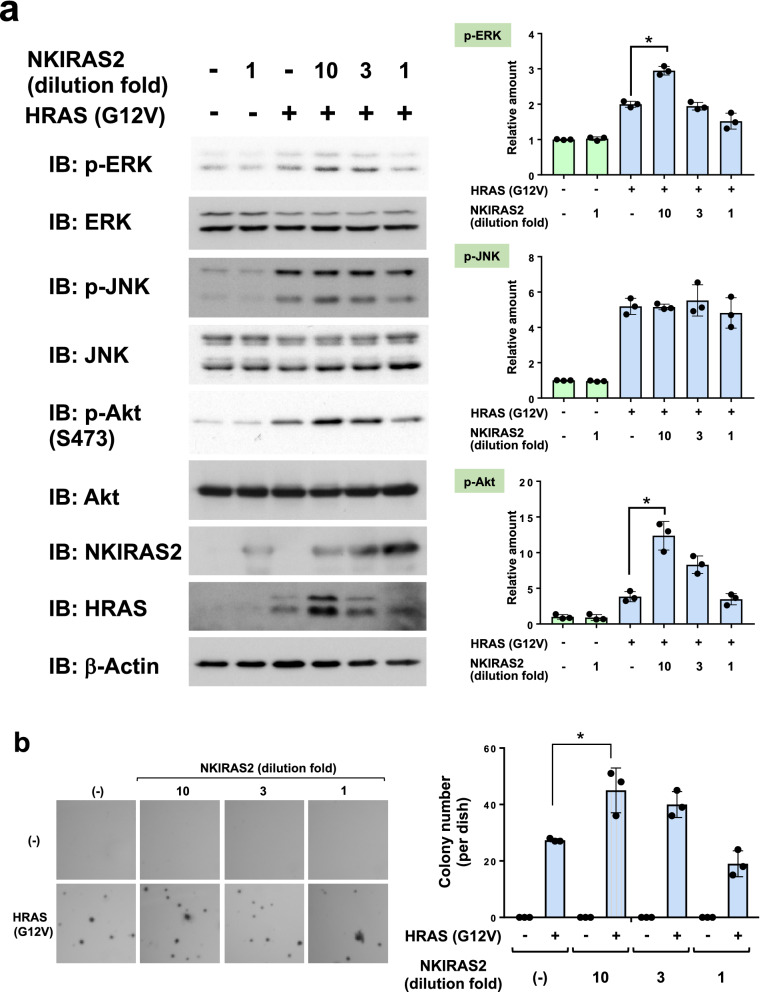
Effect of enforced expression of NKIRAS2 on HRAS (G12V)-induced cellular transformation. (**a**) NIH-3T3 cells were infected with the indicated combination of retroviruses. To analyze dose-dependent effects of NKIRAS2 on HRAS (G12V)-induced signals, the culture supernatant containing NKIRAS2 retrovirus was diluted 1-, 3- and tenfold, and then used to infect the cells. The expression of NKIRAS2 is shown using immunoblotting. The effects of various dosages of NKIRAS2 on HRAS (G12V)-induced activation of ERK, JNK, and Akt are shown. As an internal control, total amounts of ERK, JNK, and Akt are shown. The intensities of protein phosphorylation were shown in the graphs. (**b**) The infected NIH-3T3 cells as in (**a**) were utilized for a soft agar colony formation assay. The number of colonies is shown in the graph. All experiments were performed individually three times, and representative data shown in the graph. The graph shows means with the error bars indicating S.D. (n = 3), and *indicates *p* < 0.01.

In this study, we obtained two diametrically opposing experimental results on the role of NKIRAS in the mechanism of carcinogenesis (Figs. 4 and 5). To explain this discrepancy, we further analyzed the roles of NKIRAS in carcinogenesis by testing the expression dose-dependence of NKIRAS2 in Ras-provoked cellular transformation. To test this, the culture supernatant including retrovirus of NKIRAS2 was first diluted threefold and tenfold. Next, these retroviruses were infected into NIH-3T3 cells. The dilution of retrovirus successfully resulted in different intensities of expression of NKIRAS2 (Fig. 6a and Supplementary Fig. 10). To confirm whether the diluted retroviruses were enough infected into murine fibroblasts, we perform the immunostaining analysis to show the expression of FLAG-NKIRAS2. As shown in Supplementary Fig. 11, tenfold diluted NKIRAS2 retrovirus was still infected into almost all NIH-3T3 cells. As shown in Fig. 6b, tenfold diluted NKIRAS2 retrovirus exhibited the most potent efficiency on enhancing the cellular transforming activity induced by oncogenic HRAS. However, undiluted NKIRAS2 retrovirus failed to augment Ras-provoked transformation, and slightly exhibited inhibitory effect on HRAS (G12V)-provoked cellular transformation. These observations suggested that NKIRAS exhibits a biphasic bell-shaped enhancing effect on Ras-mutant-provoked oncogenic activity. Strikingly, NKIRAS2 also exhibited a similar effect on the phosphorylation of Akt at Ser473, with tenfold dilution of NKIRAS2 retrovirus exhibited most potent efficiency to augment the activation of Akt. However, higher expression of NKIRAS2 failed to enhance the activation of Akt. Unexpectedly, the expression of HRAS (G12V) was similarly enhanced by moderate expression of NKIRAS2 (Supplementary Fig. 10). Comparing with the data in Fig. 5a, it was also unexpected that moderate expression of NKIRAS2 augmented the HRAS (G12V)-induced ERK activation. Unlike the case of Akt and ERK, the activation of JNK was not affected by the enforced expression of NKIRAS2 (Fig. 6a).

## Discussion

In the current study, we first found that K15 promoter-driven enforced expression of NKIRAS2 in follicle bulges exhibits potent tumor suppressive activity against the development of skin papilloma induced by treatment with DMBA and TPA (Fig. 4). It is well understood that tumor formation is initiated from abnormal proliferation of cancer stem cells^43^. White and colleagues utilized a temporal model of tumorigenesis in vivo, and they observed tumor initiation in epidermal squamous cell carcinoma from the hair follicle stem cells niche^44^. Furthermore, several investigations reported that DMBA/TPA-induced skin tumorigenesis seems to be initiated from stem cells in follicle bulges^45,46^. Treatment with DMBA has been reported to induce mutation of the HRAS gene^35^. These reports suggested that DMBA/TPA-induced development of skin papilloma seems to be initiated from abnormal proliferation of stem cells in follicle bulges provoked by oncogenic Ras mutant. Furthermore, we found that the enforced expression of NKIRAS in follicle bulge suppresses it, suggesting that NKIRAS could function as tumor suppressor in tumor stem cells in follicle bulge.

On the other hand, we also observed that endogenous Nkiras is required for oncogenic RAS-mutant-driven cellular transformation of murine fibroblasts (Fig. 5). However, two previous reports claimed diametrically opposing conclusions about the functional directions of NKIRAS in oncogenic signaling^21,36^. Oeckinghaus and colleagues reported that NKIRAS functions as a tumor suppressor through the inhibition of RAL small GTPase by enhancing the activity of RAL GAP (GTPase activating protein)^21^. Several investigations showed that RAL contributes to tumorigenesis through the activation of JNK^47,48^. However, we showed the enforced expression of NKIRAS2 or knockdown of Nkiras2 did not affect the activation of JNK induced by oncogenic Ras mutant (Figs. 5 and 6), suggesting that NKIRAS does not contribute to tuning activation of the RAL-JNK signaling axis. On the other hand, we observed that the enforced expression or knockdown of NKIRAS2 effectively affected the phosphorylation of Akt at Ser473, which was reported to be induced by mTORC2 (Figs. 5 and 6) ^40,41^. The suppression of Akt phosphorylation by Nkiras2 knockdown was also observed in the condition without the enforced expression of oncogenic HRAS mutant. Probably, the Nkiras2 knockdown also suppressed Akt activation induced by endogenous HRAS. Although it is still unknown how mTORC2 is activated by proliferative signals including oncogenic HRAS, several reports suggested that mTORC2 activation is enhanced by the activation of AMP-dependent protein kinase and PI3K, respectively^49,50^. NKIRAS may be involved in the regulation of the mTORC2 activity through tuning AMP-dependent protein kinase and/or PI3K. Unlike the case of the classical Ras family, NKIRAS lacks the ability to interact with the RAS-binding domain of PI3K and RAF1^18^. It is worth clarifying how NKIRAS contributes to the activation of mTORC2, and these studies should provide important insights to clarify the detailed mechanism of how NKIRAS is involved in Ras-provoked cellular transformation. We also observed unexpected result that Nkiras2 knockdown enhanced the activation of ERK in the conditions with/without HRAS (G12V) (Fig. 5a). On the other hand, moderate expression of NKIRAS2 augmented the ERK activation (Fig. 6a). We cannot suitably explain these controversial observations. Although we realize the requirement of further analysis for the effect of NKIRAS on ERK pathway in future project, we conclude that ERK pathway seems to not have critical roles in facilitation by NKIRAS onto cellular transformation provoked by HRAS (G12V).

**Figure 7:**
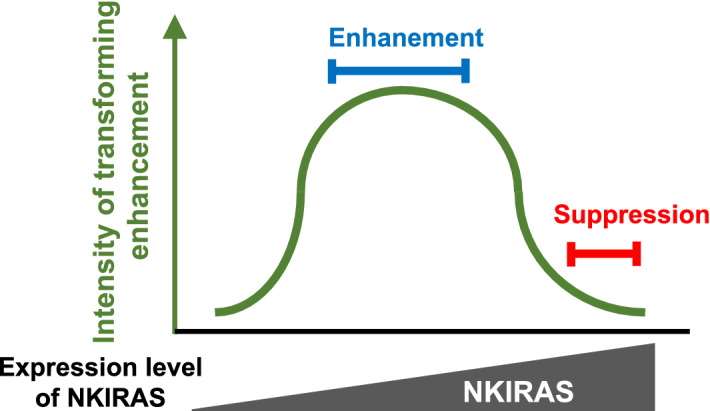
NKIRAS exhibits a “bell-shaped” expression level-dependent enhancing effect on the oncogenic Ras mutant-provoked oncogenic signals. NKIRAS2 exhibits a biphasic bell-shaped enhancing effect on RAS-mutant-provoked oncogenic activity.

We have no suitable explanation for the discrepancy that we observed in the experimental results between mice and murine fibroblasts. These opposing results may be due to not only the cell types in which NKIRAS is expressed but also the expression dosage of NKIRAS. As shown in Fig. 6, our observations suggested that the functional direction of NKIRAS against tumorigenesis is most likely determined by its expression level. Moderate enforced expression of NKIRAS2 enhanced oncogenic RAS-provoked cellular transformation, whereas an excess amount of NKIRAS2 expression turned its functional direction to a tumor suppressive phenotype (Fig. 7). In the present study, we observed that the expression of HRAS (G12V) was similarly enhanced by moderate expression of NKIRAS2 (Fig. 6a). However, we have to note that transforming activity by HRAS (G12V) is not corelated with its expression level. In our previous study, we reported that HRAS (G12V) also similarly exhibits its oncogenic activity in a bell-shaped-dependent manner^31^. According to this observation, we utilized tenfold diluted retrovirus harboring HRAS (G12V) as described in Materials and Methods, since the efficiency of cellular transformation is reduced by the expression of higher amount of HRAS (G12V). The uniqueness of the expression dosage-dependency of HRAS can be explained by a mechanism how farnesyl transferase inhibitor suppresses oncogenicity caused by the HRAS (G12V) mutant^51^. To activate downstream components from RAS such as RAF and PI3K, RAS needs to be localized on the plasma membrane mediated by post-translational modification with an isoprenyl group on its carboxy-terminal CAAX motif^52^. This lipid modification functions as an anchor linking HRAS to the plasma membrane. When HRAS (G12V)-provoked transformed cells were treated with farnesyl transferase inhibitor, HRAS (G12V) unmodified with an isoprenyl group accumulated in the cytosol but not on the plasma membrane. The cytosolic unmodified HRAS (G12V) still exhibited binding affinity to RAF and PI3K in the cytosol, and it disturbed Ras effectors from being activated on the plasma membrane. On the other hand, we also observed that HRAS (G12V) caused the enhanced expression of Nkiras2 (Fig. 5a). Similar effect was observed in the case of exogenous NKIRAS expression (Fig. 6a). As shown in Supplementary Fig. 8b, HRAS (G12V) reduced the expression of Nkiras2 mRNA. Similarly, Oeckinghaus et al. also cited several reported data, and concluded that the expression of NKIRAS2 mRNA was reduced in several cancer samples^22^. Although we still need to further analysis, HRAS (G12V) seems to enhance the expression of NKIRAS2 protein through the enhancement of protein synthesis of NKIRAS2 or its stabilization. There could be several complicated feedback mechanisms that stabilize the protein levels between HRAS and NKIRAS2 either way. As described in the introduction, NKIRAS does not possess a CAAX motif. However, to exhibit the ability of NKIRAS, it is simple to imagine that scaffolding platform for NKIRAS and signaling upstream/downstream proteins of NKIRAS should be required. Although this is our speculation, an excess amount of enforced expression of NKIRAS likely fails to position it on its suitable scaffold, and it may function as a signaling suppressor. As another possibility, we have to consider that the functional direction of NKIRAS could be determined by cellular contexts. To gain further insights into the mechanism of how NKIRAS contributes to carcinogenesis, it will be required to identify the interacting proteins of NKIRAS.

At the end, we have to point out that our current study include several problems. First, we failed to detect the mRNA expression of exogenous NKIRAS driven by K15 promoter in transgenic mice #023. Although we speculated that this result maybe due to the lower copy number of transgenes in the transgenic mice #023, we would need to optimize the condition of RT-PCR to detect the slightly expressed genes in future project. Second, since the knockdown efficiency of endogenous Nkiras2 in NIH-3T3 was insufficient, we could not completely conclude the functional importance of Nkiras2 for the cellular transformation provoked by oncogenic Ras mutant. In future projects, genome editing technique such as CRISPR-Cas9 will be useful to determine the functional significance of Nkiras2 for the carcinogenesis. Finally, we still cannot explain why we observed the bell-shaped enhancing effect by NKIRAS2 on Ras-provoked cellular transformation. The increased transforming capacity could be explained by increased HRAS expression levels. To solve this problem, we also need to analyze the mechanism how NKIRAS2 affects the activation of Akt by utilizing its sh-RNA or inhibitors. These are unsolved problems in the current study, and it will be important to clarify them to understand the functional relationship between RAS and NKIRAS in carcinogenesis.

## Conclusion

The role of NKIRAS in carcinogenesis seems to be determined by not only cellular context but also its expression level. In this study, we found that moderate enforced expression of NKIRAS facilitated oncogenic Ras to induce cellular transformation, whereas an excess amount of NKIRAS expression switched its function to a tumor suppressor.

## Supplementary Information

Supplementary Information.

Supplementary Figures.

## Abbreviations

DMBA: 7,12-Dimethylbenz(a)anthracene
EGF: Epidermal growth factor
ERK: Extracellular signal-regulated kinase
IκB: Nuclear factor of kappa light polypeptide gene enhancer in B-cells inhibitor
IκBα: Nuclear factor of kappa light polypeptide gene enhancer in B-cells inhibitor alpha
IκBβ: Nuclear factor of kappa light polypeptide gene enhancer in B-cells inhibitor beta
IKK: IκB kinase
JNK: c-Jun N-terminal kinase
mTORC1: Mammalian target of rapamycin complex 1
NF-κB: Nuclear factor kappa-light-chain-enhancer of activated B cells
PBS: Phosphate buffered saline
PDGF: Platelet-derived growth factor
PI3K: Phosphatidyl inositol-3 kinase
PVDF: Polyvinylidene difluoride
RT-PCR: Reverse transcription-polymerase chain reaction
TNFα: Tumor necrosis factor alpha
TPA: 12-*O*-tetradecanoylphorbol 13-acetate
