# Supplementary material for: K15 promoter-driven enforced expression of NKIRAS exhibits tumor suppressive activity against the development of DMBA/TPA-induced skin tumors
Source: Sci Rep. 2021 Oct 19;11:20658. doi: 10.1038/s41598-021-00200-1 (PMC8526694; doi:10.1038/s41598-021-00200-1)
Supplement: Supplementary file 1 — Supplementary Information. [file 41598_2021_200_MOESM1_ESM.pdf]

Uncropped data for immunoblot in **"K15 promoter-driven enforced expression of NKIRAS exhibits tumor suppressive activity against the development of DMBA/TPA-induced skin tumors"**

Fig. 1d

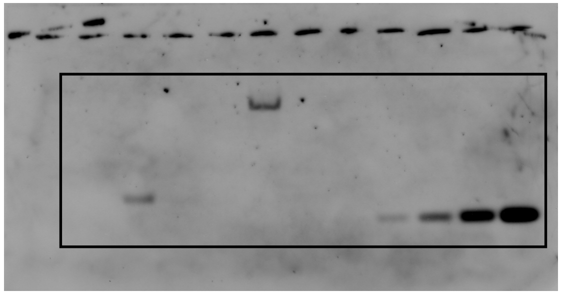

Supplementary Fig. 1

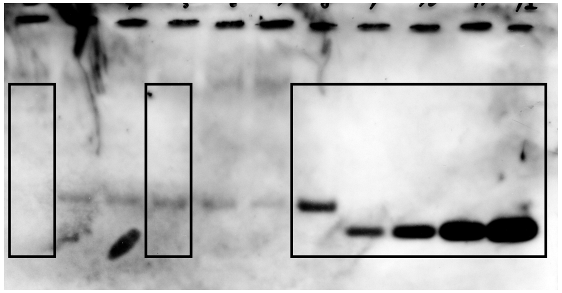

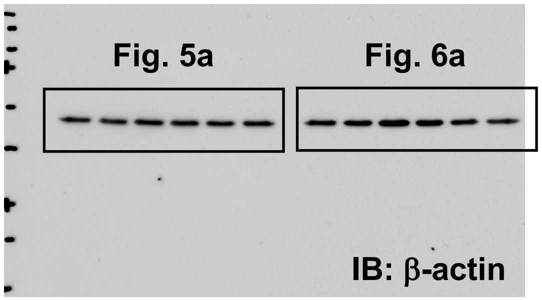

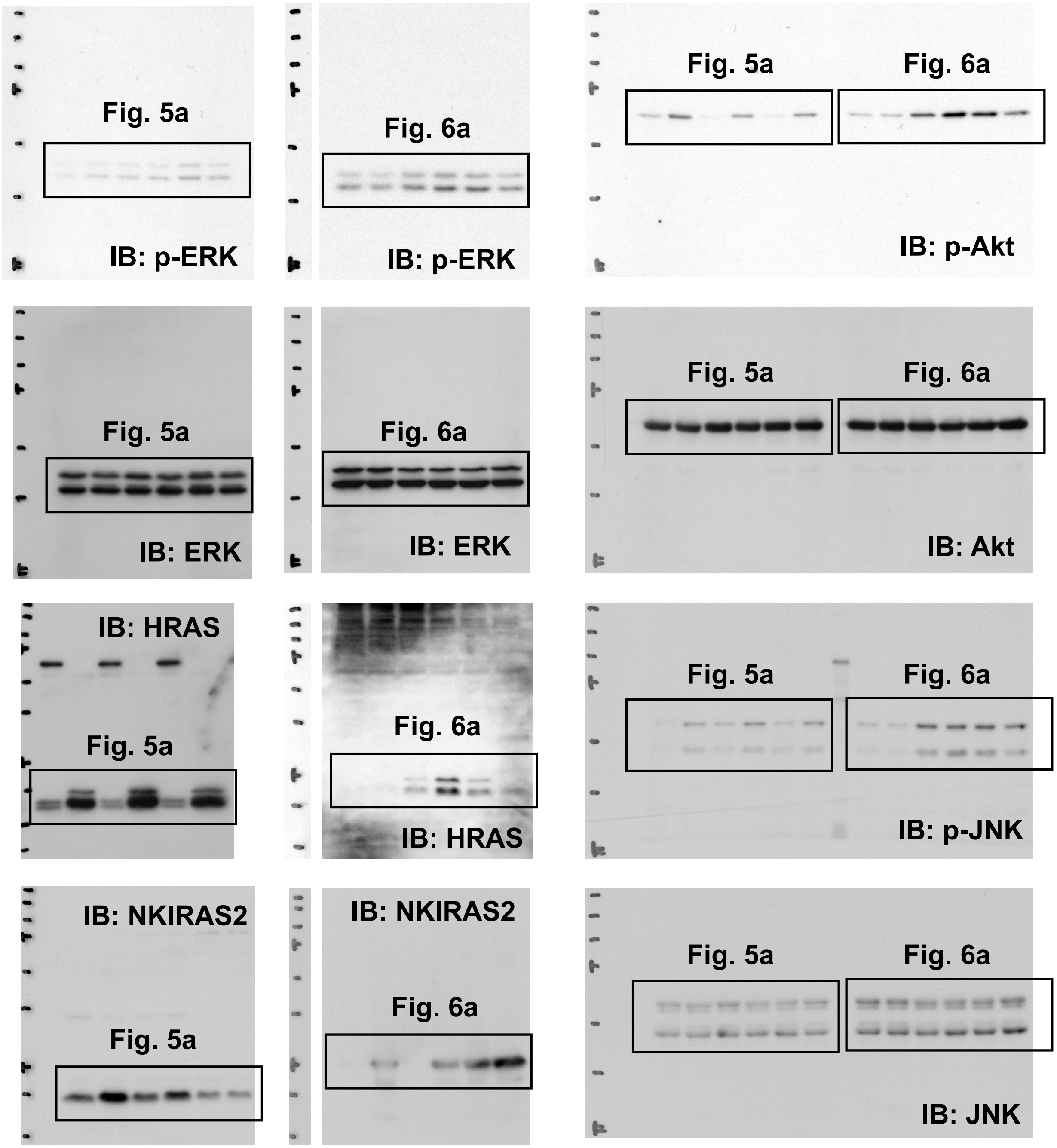

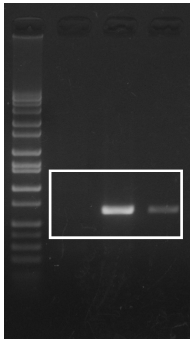

Fig. 1c

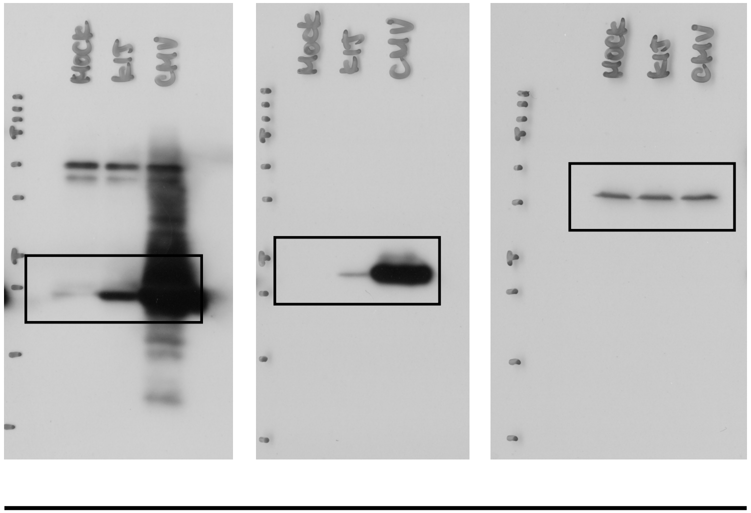

Fig. 1b

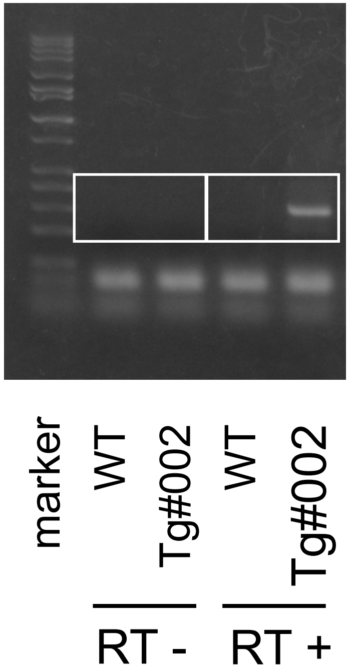

Fig. 2a
(NKIRAS2)

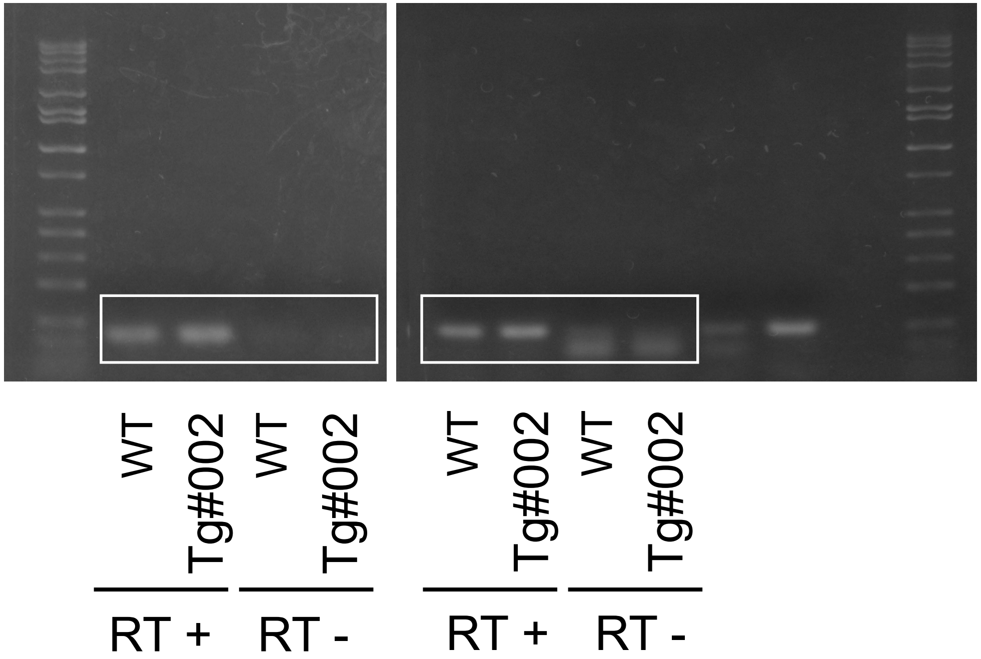

Fig. 2a
(K15 and GAPDH)
