# Supplementary material for: K15 promoter-driven enforced expression of NKIRAS exhibits tumor suppressive activity against the development of DMBA/TPA-induced skin tumors
Source: Sci Rep. 2021 Oct 19;11:20658. doi: 10.1038/s41598-021-00200-1 (PMC8526694; doi:10.1038/s41598-021-00200-1)
Supplement: Supplementary file 2 — Supplementary Figures. [file 41598_2021_200_MOESM2_ESM.pdf]

Supplementary Figures for:

K15 promoter-driven enforced expression of NKIRAS exhibits tumor suppressive activity against the development of DMBA/TPA-induced skin tumors

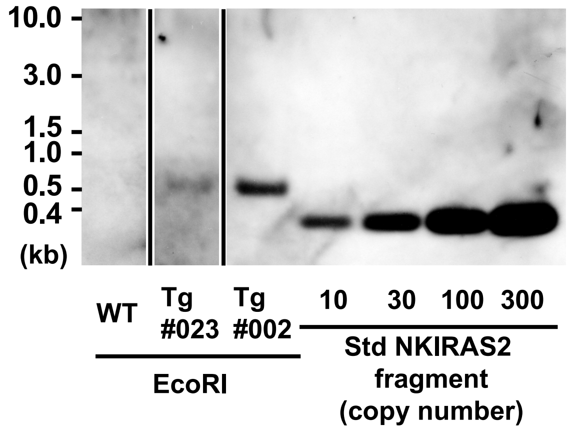

Supplementary Fig. 1 Southern blot analysis for genomic DNA prepared from NKIRAS2 transgenic mice

To determine the copy number of NKIRAS2 transgene, southern blot analysis was performed. The transgenes of FLAG-NKIRAS2 inserted into genomic DNA were quantified as about 25 and 5 copies in transgenic mice #002 and #023, respectively.

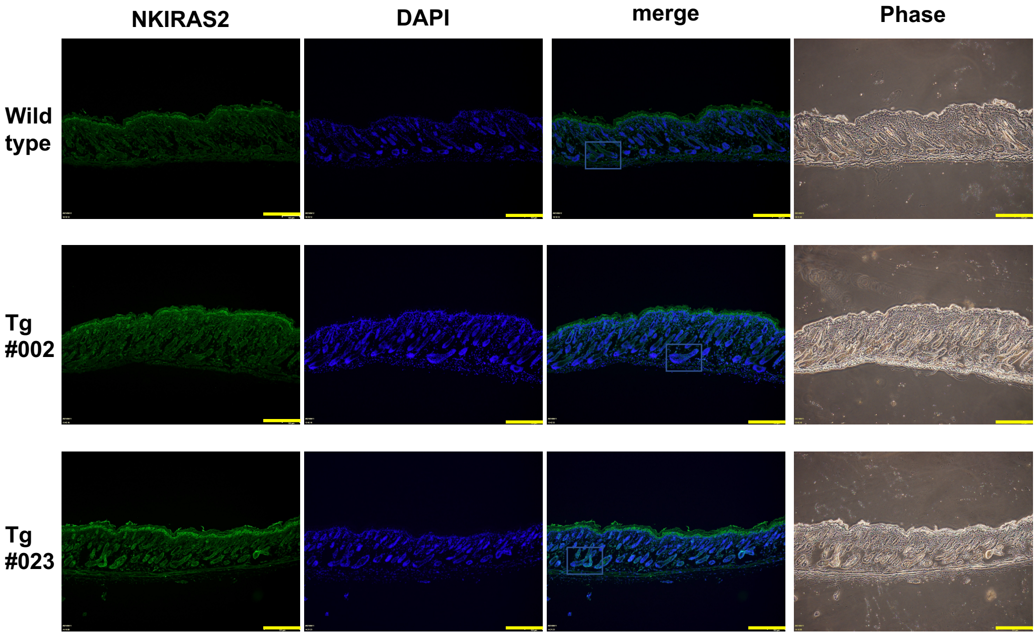

Supplementary Fig. 2 Analysis of ectopic expression of NKIRAS2 in transgenic mice by Immunofluorescence analysis (1)

Ectopic expression of NKIRAS2 driven by the K15 promoter in follicle bulges of wild type and two lines of transgenic mice is shown using immunofluorescence analysis with an anti-NKIRAS2 antibody (green). Nuclei of all cells in the epidermal section were stained with DAPI (blue). Photograph was taken by low magnifier (4.2-fold). Scale bar = 311 μm. Squared area is shown in Fig. 2b at higher magnification (40-fold).

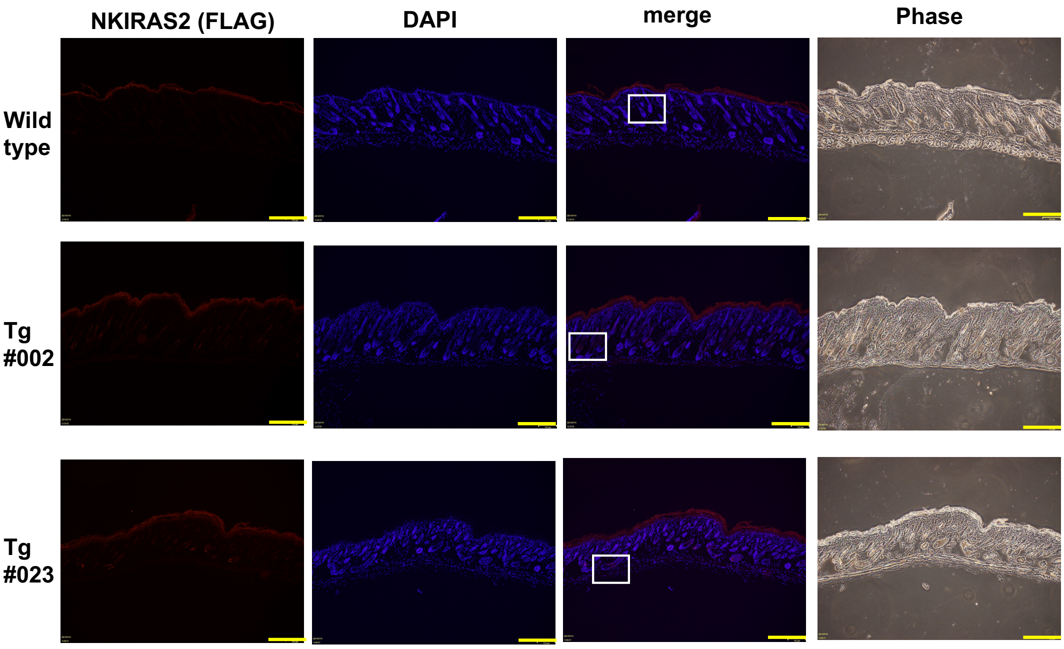

Supplementary Fig. 3 Analysis of ectopic expression of NKIRAS2 in transgenic mice by Immunofluorescence analysis (2)

To confirm that exogenous NKIRAS2 was expressed, NKIRAS2 was stained with an anti-FLAG antibody (red). Nuclei of all cells in the epidermal section were stained with DAPI (blue). Photograph was taken at low magnification (4.2-fold). Scale bar = 311 μm. Squared area is shown in Fig. 2c at higher magnification (40-fold).

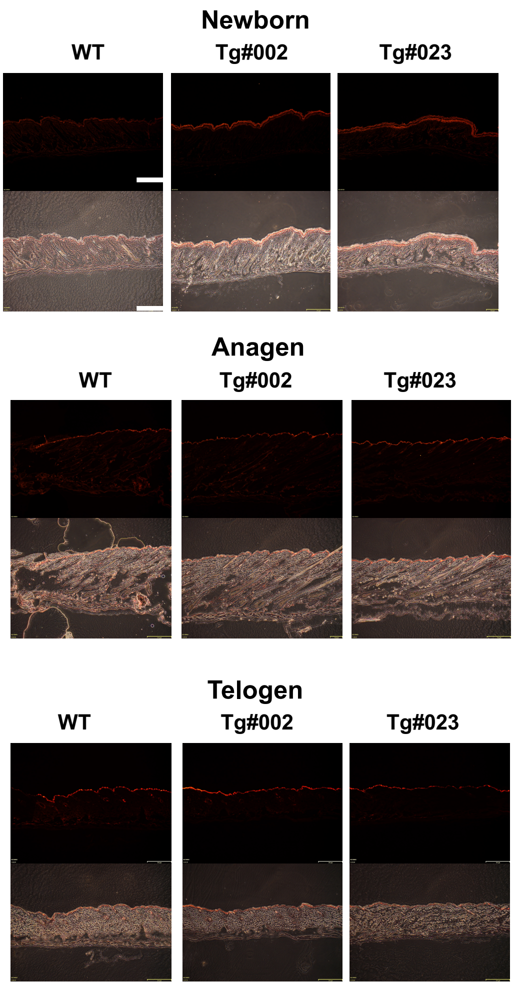

Supplementary Fig. 4 Effect of enforced expression of NKIRAS2 in follicle bulge on the expression of K10

The frozen section of skin prepared from wild type and NKIRAS2 transgenic mice were stained with anti-K10 antibody (red). Photograph was taken at low magnification (4.2-fold). Scale bar = 311 μm. Photographs at higher magnification (40-fold) were shown in Fig. 3a.

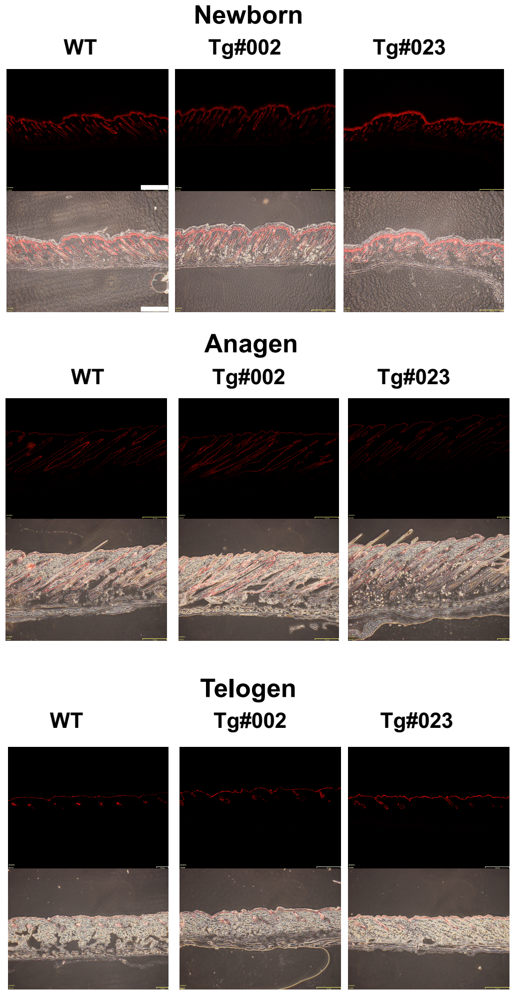

Supplementary Fig. 5 Effect of enforced expression of NKIRAS2 in follicle bulge on the expression of K14

The frozen section of skin prepared from wild type and NKIRAS2 transgenic mice were stained with anti-K14 antibody (red). Photograph was taken at low magnification (4.2-fold). Scale bar = 311 μm. Photographs at higher magnification (40-fold) were shown in Fig. 3b.

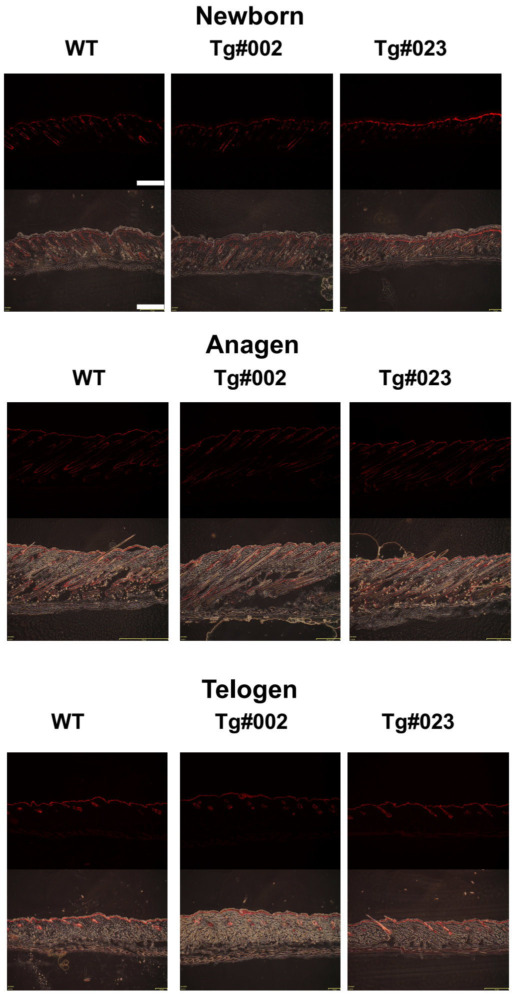

Supplementary Fig. 6 Effect of enforced expression of NKIRAS2 in follicle bulge on the expression of K15

The frozen section of skin prepared from wild type and NKIRAS2 transgenic mice were stained with anti-K15 antibody (red). Photograph was taken at low magnification (4.2-fold). Scale bar = 311 μm. Photographs at higher magnification (40-fold) were shown in Fig. 3c.

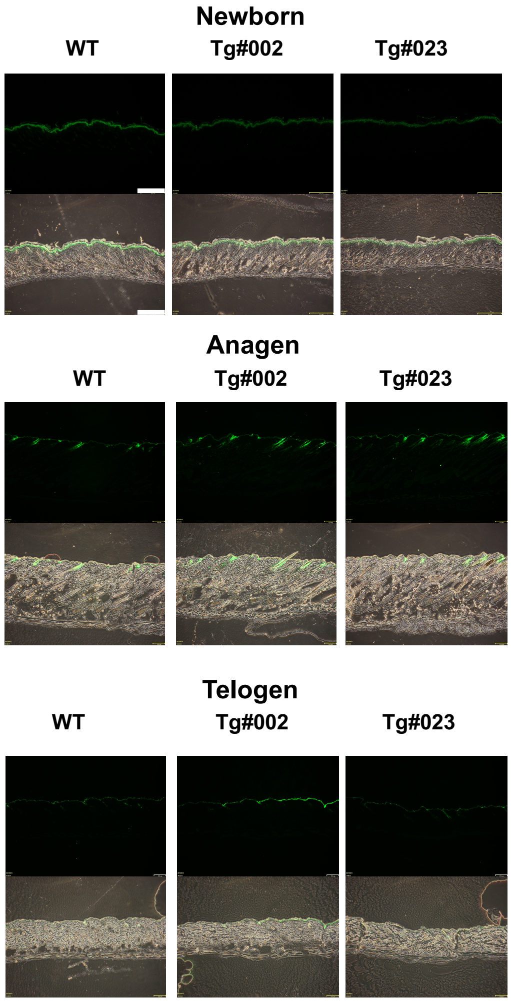

Supplementary Fig. 7 Effect of enforced expression of NKIRAS2 in follicle bulge on the expression of filaggrin

The frozen section of skin prepared from wild type and NKIRAS2 transgenic mice were stained with anti-filaggrin antibody (green). Photograph was taken at low magnification (4.2-fold). Scale bar = 311 μm. Photographs at higher magnification (40-fold) were shown in Fig. 3d.

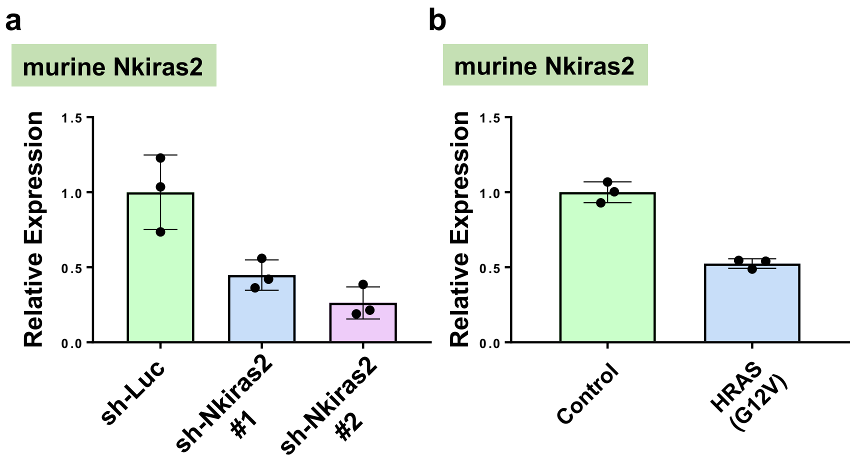

Supplementary Fig. 8 quantitative RT-PCR to evaluate the knockdown efficiency of Nkiras2 and the effect of oncogenic HRAS on the expression of Nkiras2 mRNA

a. Murine fibroblasts were infected with retroviruses harboring sh-RNA against murine Nkiras2. As control, retrovirus including sh-luciferase (sh-Luc) was infected. After puromycin selection, total RNAs were prepared, and the quantitative RT-PCR for Nkiras2 was performed. b. Effect of HRAS (G12V) on the expression of murine Nkiras2 was evaluated by the quantitative RT-PCR.

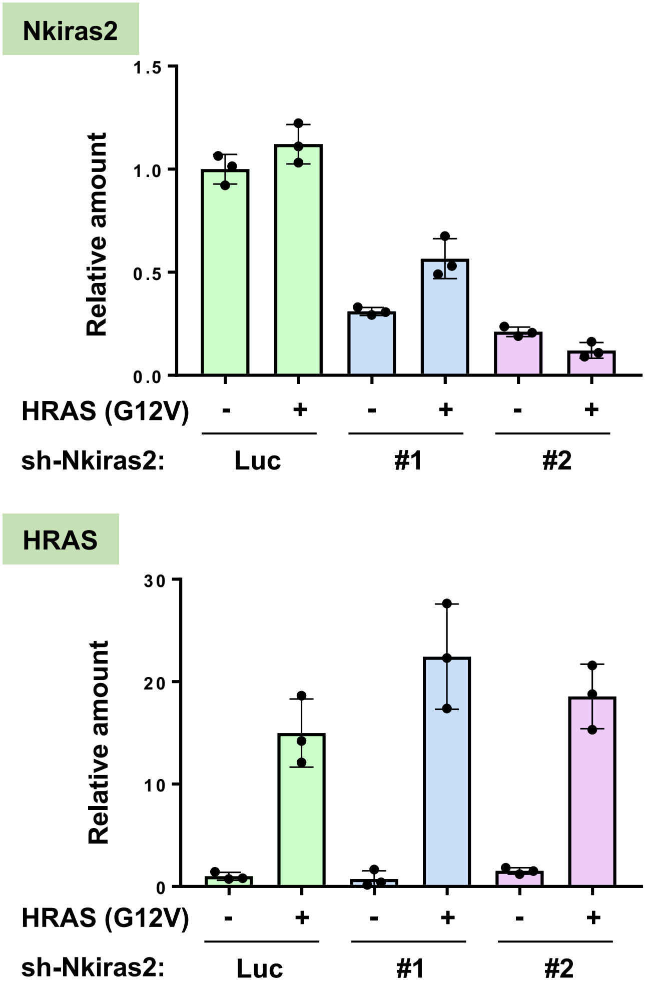

Supplementary Fig. 9 Quantification of immunoblot analysis for Nkiras2 and HRAS in NIH-3T3 cells (1)

As shown in Fig. 5a, infection of NIH-3T3 cells with two kinds of retroviruses including sh-RNA against murine Nkiras2 caused effective reductions in Nkiras2 expression. As control, retrovirus including sh-luciferase (sh-Luc; described as Luc) was infected. The band intensities of Nkiras2 and HRAS were quantified and shown in the graph. The graph shows means with the error bars indicating S.D. (n=3).

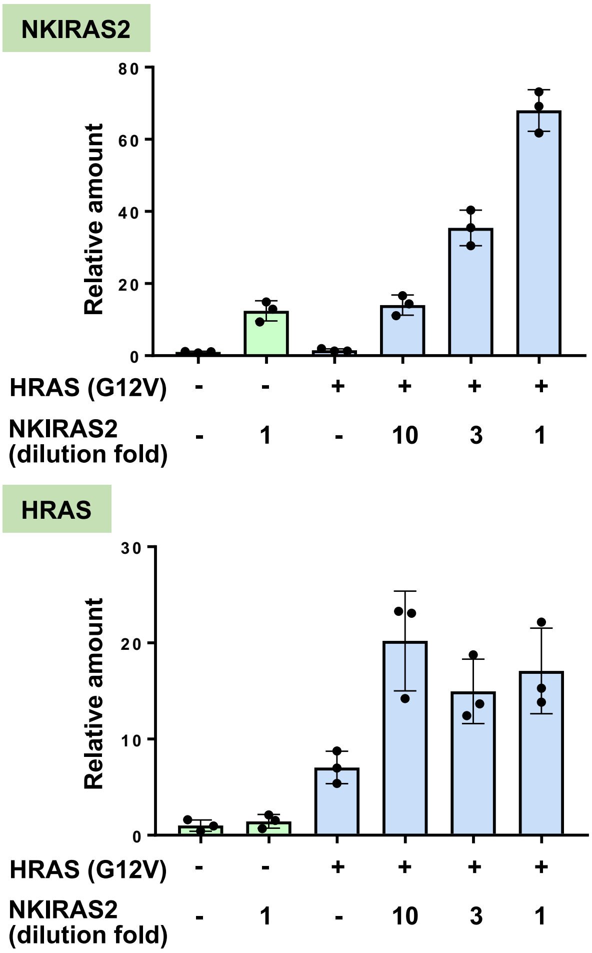

Supplementary Fig. 10 Quantification of immunoblot analysis for NKIRAS2 and HRAS in NIH-3T3 cells (2)

The culture supernatant including retrovirus of NKIRAS2 was first diluted 3-fold and 10-fold, and these retroviruses were transfected into NIH-3T3 cells as shown in Fig. 6a. The band intensities of NKIRAS2 and HRAS were quantified and shown in the graph. The graph shows means with the error bars indicating S.D. (n=3).

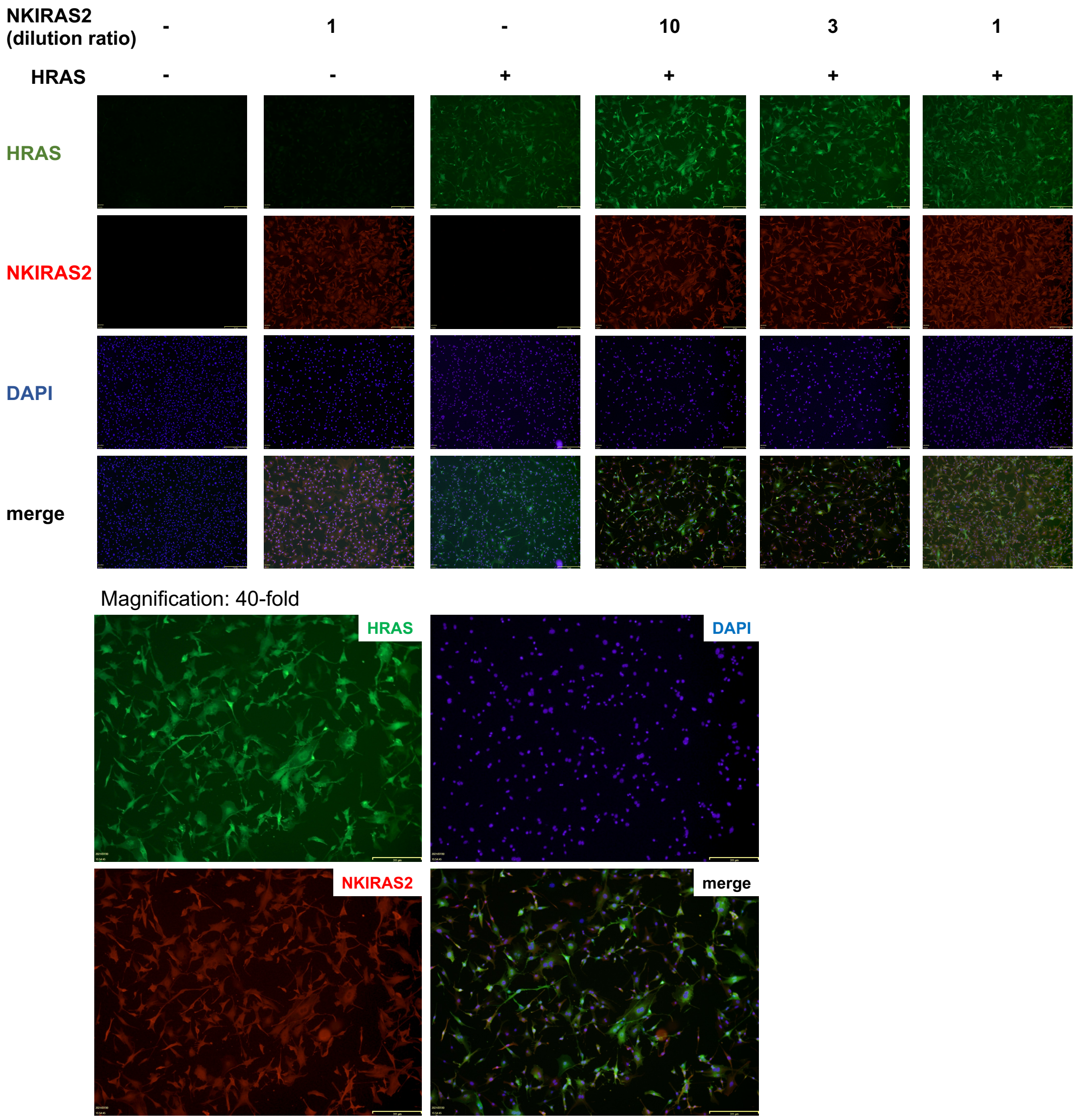

Supplementary Fig. 11 Immunofluorescence analysis of enforced expression of HRAS and NKIRAS2 in NIH-3T3 cells.

To show the infectious efficiency of retrovirus into the cells analyzed in Fig. 6, immunofluorescence analysis for HRAS and NKIRAS2 was performed. Ten-fold diluted retrovirus for NKIRAS2 exhibited almost 100% infectious efficiency as shown in the photograph taken at 40-fold magnification.
